# Maximal exercise does not increase ventilation heterogeneity in healthy trained adults

**DOI:** 10.14814/phy2.12747

**Published:** 2016-04-06

**Authors:** Jeremy P. Wrobel, Matthew J. Ellis, Kirk Kee, Christopher R. Stuart‐Andrews, Bruce R. Thompson

**Affiliations:** ^1^ Department of MedicineMonash UniversityMelbourneAustralia; ^2^ Allergy, Immunology & Respiratory MedicineThe AlfredMelbourneAustralia; ^3^ Department of Respiratory MedicineFiona Stanley HospitalPerthAustralia

**Keywords:** Cardiopulmonary exercise test, hypoxemia, lung clearance index, multiple breath nitrogen washout, ventilation inhomogeneity

## Abstract

The effect of exercise on ventilation heterogeneity has not been investigated. We hypothesized that a maximal exercise bout would increase ventilation heterogeneity. We also hypothesized that increased ventilation heterogeneity would be associated with exercise‐induced arterial hypoxemia (EIAH). Healthy trained adult males were prospectively assessed for ventilation heterogeneity using lung clearance index (LCI), S_cond_, and S_acin_ at baseline, postexercise and at recovery, using the multiple breath nitrogen washout technique. The maximal exercise bout consisted of a maximal, incremental cardiopulmonary exercise test at 25 watt increments. Eighteen subjects were recruited with mean ± SD age of 35 ± 9 years. There were no significant changes in LCI, S_cond_, or S_acin_ following exercise or at recovery. While there was an overall reduction in SpO_2_ with exercise (99.3 ± 1 to 93.7 ± 3%, *P *<* *0.0001), the reduction in SpO_2_ was not associated with changes in LCI, S_cond_ or S_acin_. Ventilation heterogeneity is not increased following a maximal exercise bout in healthy trained adults. Furthermore, EIAH is not associated with changes in ventilation heterogeneity in healthy trained adults.

## Introduction

Alveolar ventilation/perfusion ${\dot{V}}_{A}/\dot{Q}$ inequality appears to occur in all subjects with at least moderate exercise, however, the underlying mechanisms for this have not been clearly established (Dempsey and Wagner 1999; Hopkins 2006). Nevertheless, potential causes for this include subtle anatomical factors that limit the capacity of airways or pulmonary vessels to cope with increased flow, changes in airway or vascular tone, airway secretions, and/or mild interstitial pulmonary edema (Podolsky et al. 1996; Dempsey and Wagner 1999; McKenzie et al. 2005; Hopkins 2006; Zavorsky et al. 2006a; Burnham et al. 2009). These postulated mechanisms may give rise to changes in ventilation and/or perfusion heterogeneity (Wagner 1992). In support of this, magnetic resonance imaging (MRI) derived perfusion heterogeneity has been demonstrated to increase following prolonged exercise in healthy trained subjects (Burnham et al. 2009). In contrast, whether ventilation heterogeneity increases with exercise remains speculative (Hopkins 2006; Burnham et al. 2009).

Additionally, among healthy trained individuals, there is a subset that experience exercise‐induced arterial hypoxemia (EIAH); however, the mechanisms underpinning this are also not clear (Gavin and Stager 1999; Rice et al. 1999; Calbet et al. 2008). Studies using the multiple inert gas elimination technique (MIGET) have demonstrated that both ${\dot{V}}_{A}/\dot{Q}$ inequality and diffusion limitation of oxygen (O_2_) equally contribute to reduced oxygenation with exercise (Hopkins et al. 1994; Dempsey and Wagner 1999; Rice et al. 1999; Hopkins 2006). However, changes in ${\dot{V}}_{A}/\dot{Q}$ inequality alone, without diffusion limitation, are not sufficient to cause EIAH (Dempsey and Wagner 1999; Hopkins 2006). While physiological studies continue to explore the role of diffusion limitation in EIAH, the relationship between ventilation heterogeneity and EIAH has not been examined.

Overall, ventilation heterogeneity within the lung can be assessed with the lung clearance index (LCI), calculated from the multiple breath nitrogen washout (MBNW) technique. Furthermore, regional ventilation heterogeneity indices can be calculated for the small conductive airways (S_cond_) and the distal acinar regions (S_acin_) of the lung (Robinson et al. 2013). Studies have demonstrated increased S_cond_ and/or S_acin_ in asthma, chronic obstructive pulmonary disease, bronchiolitis obliterans following lung transplantation and chronic heart failure, with evidence that it may be a more sensitive marker for airway disease than spirometry (Verbanck et al. 2004, 2006; Thompson et al. 2014; Kee et al. 2015). The sensitivity of S_cond_ and S_acin_ suggest a potential role for using these measures of ventilation heterogeneity to assess asthma control (Bourdin et al. 2006; Downie et al. 2007).

With mounting evidence that MBNW is a sensitive marker of ventilation heterogeneity, we sought to use this technique to determine whether a single maximal exercise bout would lead to increased ventilation heterogeneity. In addition, we sought to determine whether increased ventilation heterogeneity was associated with EIAH.

## Materials and methods

### Subjects

Healthy trained male participants from local sports groups were prospectively recruited. We chose “trained” adults for four main reasons: (1) to reduce heterogeneity of study participants; (2) to ensure participants were motivated to achieve VO_2_max rather than resulting in a submaximal test; (3) to enable standardization of the incremental maximal test; and (4) to minimize the risk of recruiting participants with occult cardiac or respiratory disease. Only male participants were included as gender differences in respiratory function and gas exchange (Hopkins and Harms 2004; Harms 2006; Harms and Rosenkranz 2008) may have confounded the analysis. Participants met the inclusion criteria if they were male, older than 18 years, free from cardiac or respiratory illness, and performed vigorous aerobic exercise more than three times per week on a regular basis. Participants were excluded if they were current or ex‐smokers, had a history of adult or childhood asthma, or used inhaler medications. The study was approved by our institution's Human Ethics Committee and all participants provided written and informed consent.

### Study design

**Figure 1:**
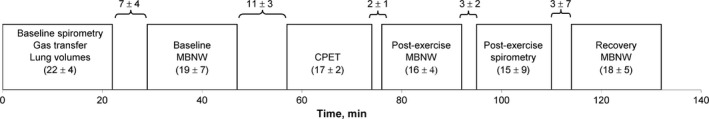
Timeline of the study protocol. Values are mean minutes ± SD for each investigation, including set‐up times. Postexercise spirometry includes post‐bronchodilator spirometry. CPET, cardiopulmonary exercise test; MBNW, multiple breath nitrogen washout.

All subjects completed the study protocol as depicted in Figure 1. In brief, subjects underwent baseline respiratory function assessment including pre‐bronchodilator spirometry, gas transfer, and lung volumes (body plethysmography). Measurements of lung function were obtained using standard equipment (Medgraphics Platinum series, Minnesota) in accordance with the ATS/ERS guidelines (Macintyre et al. 2005; Miller et al. 2005; Wanger et al. 2005). Prediction equations for spirometry, gas transfer, and lung volumes were those of Knudson (Knudson et al. 1983), Roca (Roca et al. 1990) and Goldman (Goldman and Becklake 1959), respectively. We utilized the prediction equations from Knudson et al. and Goldman & Becklake as it is our experience that is more closely matches the Australian population than the more recent reference equations of Quanjer et al. (2012) (Quanjer et al. 2012) and Quanjer et al. (1993) (Quanjer et al. 1993), respectively.

Subjects then performed a baseline MBNW (see below) followed by a maximal stage 1 incremental cardiopulmonary exercise test (CPET) (American Thoracic Society and American College of Chest Physicians, 2003) on standard cycle ergometer (Sensormedics Vmax Spectra, California). This consisted of a protocol of 25 watt increments every minute and the test was terminated when the workload could not be maintained above 50 revolutions per minute. Pulse oximetry (SpO_2_) was recorded throughout the CPET (MasimoSet Rainbow Radical 7 signal extraction pulse oximeter, Irvine, California) consistent with previous studies (Macnutt et al. 2007) and the oximeter visual waveform was monitored to ensure adequate peripheral perfusion. CPET was immediately followed by a postexercise MBNW and repeat spirometry with pre‐ and post‐bronchodilator (three inhalations of salbutamol 100 mcg administered via a spacer) assessment to exclude exercise induced bronchoconstriction or reversible airways obstruction. Exercise‐induced bronchoconstriction was defined as a reduction in FEV_1_ of 10% following exercise compared with baseline FEV_1_, whereas reversible airways obstruction was defined as an increase in FEV_1_ and/or FVC of 200 mL and 12% following inhaled bronchodilator (Pellegrino et al. 2005) in comparison with the postexercise pre‐bronchodilator spirometry. Finally, participants performed a recovery MBNW 30 min following completion of the CPET.

### Multiple breath nitrogen washout

Ventilation heterogeneity was assessed by the MBNW technique. Instrumentation consisted of a bag in box system where flow was measured using a precalibrated pneumotachograph (Fleish type, flow range 0–5 L sec^−1^) and N_2_ measured continuously at the patient's mouth (AU9240‐4032, Medgraphics Corporation, Minnesota).

Overall, lung ventilation heterogeneity was assessed by the LCI, defined as the number of FRC turnovers (calculated as the ratio of the cumulative expired volume to the FRC volume) required to reduce alveolar nitrogen concentration to less than 2% (Robinson et al. 2013). Ventilation heterogeneity was also measured for the small conductive regions (S_cond_) and distal acinar regions (S_acin_) of the lungs.

Analysis and underlying theory of the MBNW test have been documented elsewhere (Crawford et al. 1985; Verbanck et al. 1997, 1998, 2004; Stuart‐Andrews et al. 2011; Robinson et al. 2013)**.** Briefly, the MBNW measures the change in slope of the nitrogen alveolar plateau (Sn) following consecutive 100% O_2_ breathing using a 1 L tidal volume, until nitrogen was washed out to below 2%. The rate of rise of Sn versus lung turnover plot between turnovers 1.5 and 6 is a measure of conductive ventilation heterogeneity (S_cond_) proximal to the acinar entrance. As the slope of the alveolar plateau has a contribution of both acinar and conductive components of ventilatory heterogeneity, S_acin_ is calculated as the normalized slope of the first breath minus the contribution of S_cond_ to the first breath. MBNW data were analyzed using an automated process previously described by our group (Stuart‐Andrews et al. 2011). Each MBNW comprised of three successive washout maneuvers with rest of 4 min between assessments to enable normalization of respiratory gas concentrations.

### Definition of EIAH

Dempsey and Wagner have previously used SaO_2_ to define EIAH as mild (93 ≤ SaO_2_ < 95%), moderate (88 ≤ SaO_2_ < 93%), or severe (SaO_2_ < 88%) (Dempsey and Wagner 1999). As we employ SpO_2_, which is less precise than SaO_2_ (Yamaya et al. 2002; Kolb et al. 2004), we have restricted the definition of EIAH in this study to moderate and severe, defined as SpO_2_ < 93%.

### Statistical analysis

Statistical analysis comparing pre‐exercise, postexercise, and recovery MBNW were performed using repeated measures ANOVA using a lower bound correction for violations of sphericity. Pearson's correlation coefficients were determined for the percentage change in SpO_2_ and changes in both S_cond_ and S_acin_ following exercise. Pre‐ and postexercise comparisons were performed using the paired samples *t* test. Comparisons between subjects with EIAH and without EIAH were performed using the independent samples *t* test. For all analysis, two‐tailed *P *<* *0.05 was considered statistically significant. Data are presented as means ± SEM for S_cond_ and S_acin_, and means ± SD for all other parameters. Statistical analysis was performed using PASW (SPSS) Statistics 18.0 (IBM Corporation, NY).

## Results

### Study participants

Eighteen participants were recruited and completed the protocol. All subjects had normal indices for baseline respiratory function assessment and CPET. Baseline demographics and physiologic parameters are included in Table 1. The mean VO_2_max for the cohort was 49 ± 6 mL min^−1^ kg^−1^ and the maximum workload achieved was 369 ± 47 W. Functional residual capacity obtained from body plethysmography was comparable with MBNW results, measuring 3.9 ± 0 L and 3.8 ± 1 L (*P *=* *0.273), respectively.

**Table 1:** Baseline demographics and physiologic parameters

	All (*n* = 18)	No EIAH (*n* = 11)	EIAH (*n* = 7)	*P* a
Age, years	35 ± 9	34 ± 9	37 ± 9	0.630
Height, cm	181 ± 5	182 ± 5	179 ± 4	0.223
Weight, kg	78 ± 5	78 ± 5	79 ± 5	0.612
BMI, kg m^−2^	24 ± 2	24 ± 1	25 ± 2	0.164
FEV_1_, % predicted	104 ± 8	107 ± 6	98 ± 7	0.013
FVC, % predicted	111 ± 9	114 ± 7	105 ± 11	0.042
FEV_1_/FVC, %	78 ± 5	77 ± 5	78 ± 4	0.864
TLCO, % predicted	113 ± 10	116 ± 10	107 ± 8	0.104
FRC, L (body plethysmography)	3.9 ± 0	3.9 ± 0	3.7 ± 0	0.472
FRC, L (MBNW)	3.8 ± 1	3.8 ± 1	3.4 ± 0	0.156
TLC, % predicted	108 ± 9	112 ± 7	100 ± 6	0.005
RV, % predicted	89 ± 16	93 ± 17	79 ± 7	0.106
RV/TLC, %	22 ± 3	23 ± 4	22 ± 1	0.471
VO_2_max, mL min^−1^ kg^−1^	49 ± 6	51 ± 6	47 ± 6	0.177
Workload, W	369 ± 47	373 ± 47	364 ± 50	0.720
Wmax, % predicted	161 ± 16	158 ± 16	164 ± 16	0.469
Peak SpO_2_, %	99.3 ± 1	99 ± 1	99 ± 1	0.307
Minimum SpO_2_, %	93.7 ± 3	96 ± 1	91 ± 1	<0.001
Postexercise Change FEV_1_, %^b^	3.3 ± 3.0	1.9 ± 4	5.4 ± 2	0.057
Post BD Change FEV_1_, %^c^	2.7 ± 2.9	3.2 ± 3	2.0 ± 2	0.416

Values are means ± SD. BD, bronchodilator; EIAH, exercise‐induced arterial hypoxemia; FEV1, forced expiratory ratio in 1 sec; FRC, functional residual capacity; FVC, forced vital capacity; MBNW, multiple breath nitrogen washout; RV, residual volume; TLC, total lung capacity; TLCO, transfer factor for carbon monoxide; VO_2_max, maximal oxygen uptake; Wmax, maximum workload achieved.

a *P* values compare No EIAH versus EIAH.

b Compared with baseline spirometry.

c Compared with postexercise, pre‐bronchodilator spirometry.

Seven of 18 participants (39%) had at least moderate EIAH (SpO_2_ < 93%). Participants with EIAH had lower FEV_1_, forced vital capacity, and total lung capacity at baseline (Table 1) compared to subjects with no EIAH. No subjects had evidence of exercise‐induced bronchoconstriction or a significant bronchodilator response on postexercise spirometry to suggest undiagnosed asthma (Pellegrino et al. 2005).

### Ventilation heterogeneity

**Figure 2:**
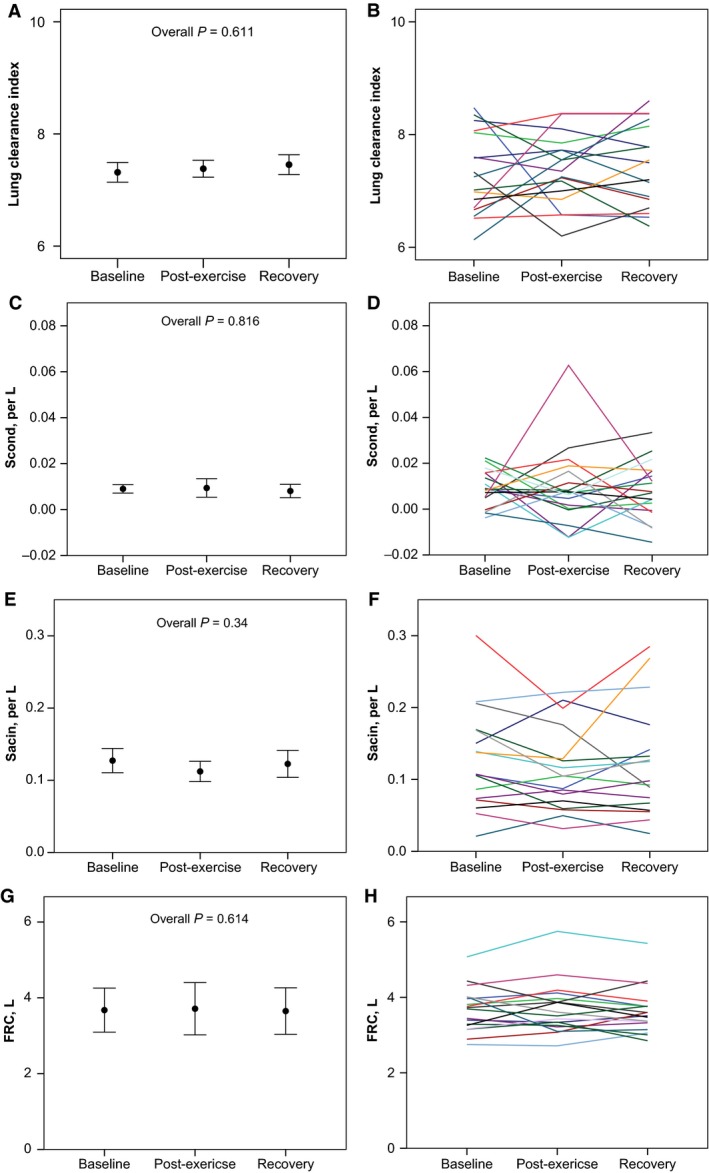
There were no significant differences across baseline, postexercise and recovery for LCI (panels A–B), S_cond_ (panels C–D), S_acin_ (panels E–F), or FRC (panels G–H). Error bars are means ± SEM for S_cond_ and S_acin_, and means ± SD for LCI and FRC. Individual responses are displayed in panels B, D, F, and H. FRC, functional residual capacity; LCI, lung clearance index.

There were no significant changes in LCI, S_cond_, or S_acin_ across baseline, postexercise, and recovery MBNW as depicted in Figure 2. Similarly, there was no change in FRC across pre‐exercise, postexercise, and recovery MBNW.

### Exercise‐induced arterial hypoxemia

**Figure 3:**
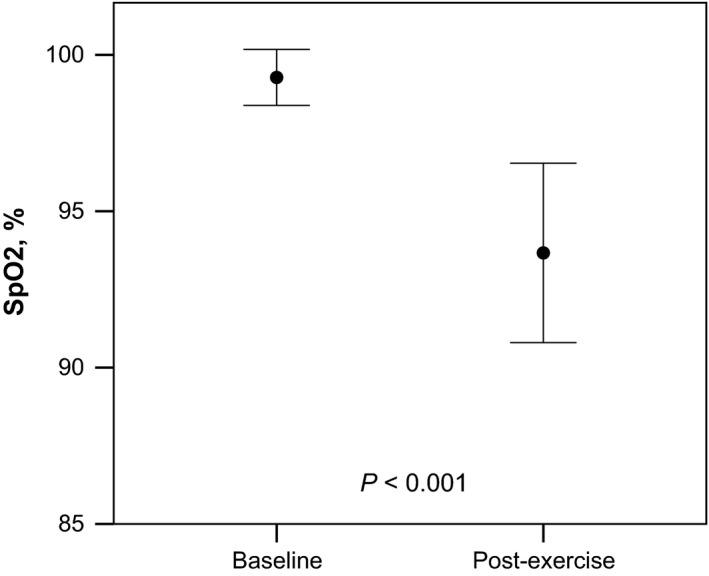
Exercise‐induced changes in SpO_2_. Error bars are means ± SD.

**Figure 4:**
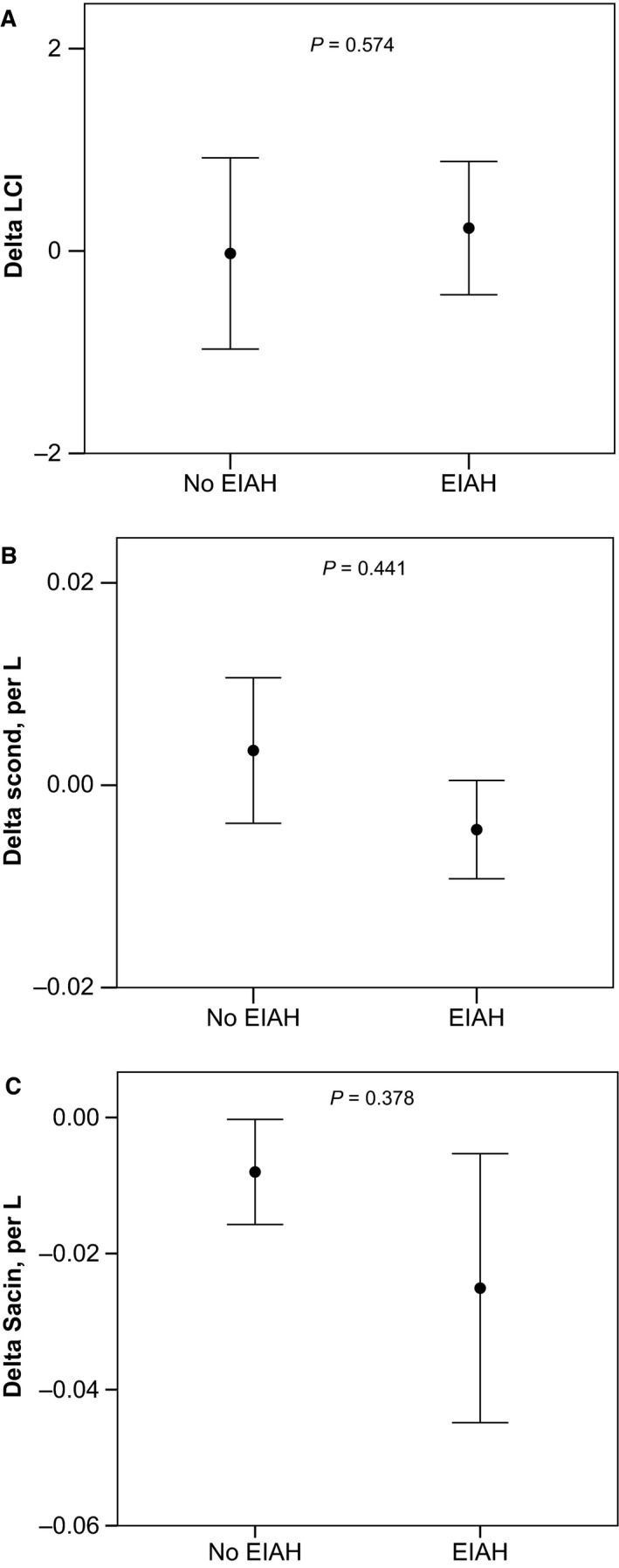
Comparing subjects with and without EIAH, there were no significant differences in LCI (panel A), S_cond_ (panel B), or S_acin_ (panel C). Error bars are means ± SD for LCI and means ± SEM. EIAH, exercise‐induced arterial hypoxemia.

**Figure 5:**
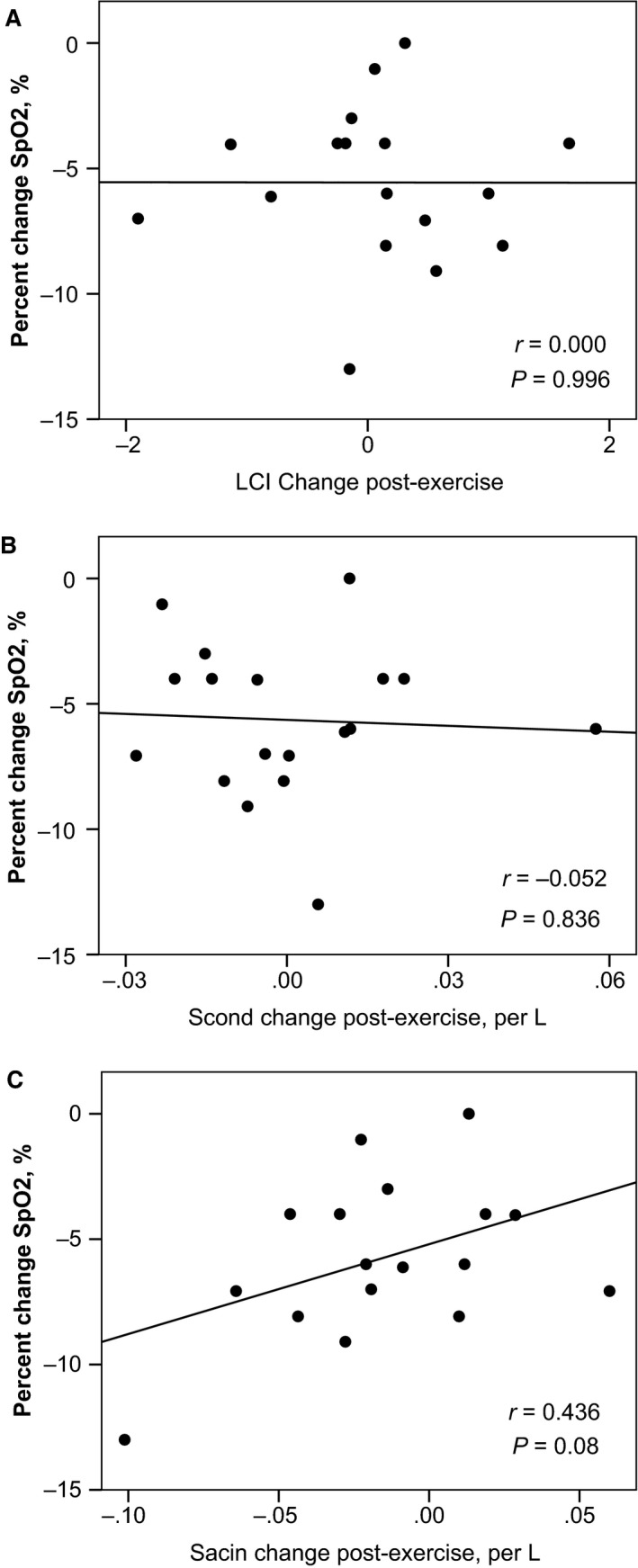
Plots of percentage change in SpO_2_ versus the postexercise changes in LCI (panel A), S_cond_ (panel B), and S_acin_ (panel C).

Overall, end‐tidal carbon dioxide (CO_2_) was not significantly different at baseline and end‐exercise (38 ± 3 vs. 40 ± 5 mm Hg, respectively, *P *=* *0.104). However, there was a significant fall in SpO_2_ during exercise (99.3 ± 1 to 93.7 ± 3%, *P *<* *0.0001, Fig. 3). There were no differences in LCI**,** S_cond_, or S_acin_ among subjects with and without EIAH (Fig. 4). Finally, the percentage change in SpO_2_ with exercise was not significantly correlated with the exercise‐related changes in LCI, S_cond_, or S_acin_ as displayed in Figure 5.

### Multiple breath nitrogen washout and respiratory flow rates

In order to determine whether the postexercise flow rates impacted upon the MBNW results, we separately analyzed the three washout maneuvers comprising each participant's postexercise MBNW result (Table 2). As expected, there was a significant reduction in the respiratory rate across each washout maneuvers from 1 to 3 (*P *<* *0.001) and a corresponding increase in the mean inspiratory and expiratory flow rates (*P *<* *0.001). Nevertheless, there was no significant change in S_cond_ or S_acin_ across these three maneuvers (*P *=* *0.248 and *P *=* *0.457, respectively). Furthermore, there were no significant relationships between measures of ventilation heterogeneity and mean inspiratory or expiratory flow rates across any of the postexercise maneuvers.

**Table 2:** Post‐exercise MBNW maneuver results and flow rates

	Maneuver 1	Maneuver 2	Maneuver 3	*P*
LCI	7.71 ± 1.31	7.54 ± 0.98	8.15 ± 1.34	0.128
S_cond_, L^−1^	0.016 ± 0.02	0.01 ± 0.03	0.002 ± 0.02	0.248
S_acin_, L^−1^	0.145 ± 0.11	0.141 ± 0.05	0.17 ± 0.1	0.457
Respiratory rate, min^−1^	23 ± 4	18 ± 3	16 ± 3	<0.001
Mean inspiratory flow, L sec^−1^	0.96 ± 0.18	0.67 ± 0.11	0.59 ± 0.11	<0.001
Mean expiratory flow, L sec^−1^	0.85 ± 0.2	0.69 ± 0.23	0.62 ± 0.18	<0.001

Values are mean ± SD. *P* values calculated using repeated measures analysis of variance. LCI, lung clearance index; MBNW, multiple breath nitrogen washout; S_acin_, acinar ventilation heterogeneity index; S_cond_, conductive ventilation heterogeneity index.

## Discussion

### Ventilation heterogeneity and exercise

${\dot{V}}_{A}/\dot{Q}$ inequality increases with significant exertion and this is likely to result from a multitude of factors. It had been postulated that increased ventilation heterogeneity may be a contributing factor (Hopkins 2006). However, this study demonstrates that a single incremental maximal exercise bout does not significantly increase ventilation heterogeneity as measured by MBNW in healthy trained males. In addition, there were no significant changes in ventilation heterogeneity upon recovery from a single incremental maximal exercise bout.

In contrast, Burnham and colleagues (Burnham et al. 2009) used MRI arterial spin labeling to demonstrate increased pulmonary perfusion heterogeneity after prolonged heavy exertion. They were also able to demonstrate a significant positive relationship between MRI pulmonary perfusion heterogeneity and MIGET perfusion heterogeneity. While Burnham and colleagues argue that increased pulmonary perfusion heterogeneity supports the notion of pulmonary edema as the mechanism for exercise‐induced increases in ${\dot{V}}_{A}/\dot{Q}$ inequality, they accept that their study falls short of direct evidence.

Although we have not investigated for the presence of exercise‐induced pulmonary edema, our results suggest that if transient pulmonary edema is the mechanism for EIAH as has been suggested, then transient pulmonary edema does not increase ventilation heterogeneity in healthy trained subjects. Similarly, Kee and colleagues (Kee et al. 2015) did not identify any differences in ventilation heterogeneity in association with the rostral fluid shifts of chronic heart failure patients in moving from seated to the supine position, which supports the notion that acute or transient pulmonary edema does not increase ventilation heterogeneity.

### Ventilation heterogeneity and EIAH

It remains unclear as to why only some subjects experience EIAH. In our study, EIAH was prevalent with 39% of subjects experiencing EIAH (defined as SpO_2_ < 93%). This prevalence of EIAH is similar to that previously described in healthy endurance athletes (Powers et al. 1988). EIAH in our cohort was not explained by exercise intensity as subjects with and without EIAH achieved similar maximal workloads and VO_2_max.

We had postulated that increased ventilation heterogeneity may contribute to EIAH, but we were unable to demonstrate any differences in LCI, S_cond_, or S_acin_ after a maximal exercise bout among subjects with EIAH compared to those without. Furthermore, there were no significant correlations between the percentage change in SpO_2_ and changes in ventilation heterogeneity.

An inadequate hyperventilatory response to exercise has been suggested to contribute to EIAH (Harms and Stager 1995; Gavin et al. 1998). However, the exercise‐induced change in end‐tidal CO_2_ was not significantly different between subjects with and without EIAH (−1.3 ± 4 vs. −2.4 ± 5, respectively, *P *=* *0.615) suggesting that this did not contribute to EIAH in this study.

The development of interstitial pulmonary edema during exercise has been postulated to be one of the major mechanisms of EIAH, which contributes to both ${\dot{V}}_{A}/\dot{Q}$ inequality and diffusion limitation. While radiological evidence of increased interstitial pulmonary edema has been demonstrated following sustained, heavy exercise in some studies (McKechnie et al. 1979; Caillaud et al. 1995; Anholm et al. 1999; McKenzie et al. 2005; Zavorsky et al. 2006a), other studies have not confirmed this relationship (Gallagher et al. 1988; Manier et al. 1999; Macnutt et al. 2007). Zavorsky reviewed the literature on pulmonary edema following exercise and demonstrated that pulmonary edema was more likely to occur following an exhaustive sustained maximal exercise effort rather than a prolonged effort (Zavorsky 2007). Furthermore, repeated near‐maximal exercise does not worsen PaO_2_ or the alveolar‐arterial oxygen gradient (Zavorsky et al. 2006b). The relationship between pulmonary perfusion heterogeneity and pulmonary edema following heavy exertion is yet to be established.

### Limitations

There are several limitations of this study that warrant attention. First, without arterial blood gas sampling, the impact of changes in ventilation heterogeneity on the PaO_2_ and alveolar‐arterial oxygen gradient cannot be accurately established. Nevertheless, if EIAH is defined as “a threat to O_2_ transport” then measuring SpO_2_, as we have done, is an appropriate measure (Dempsey and Wagner 1999). Secondly, although the postexercise MBNW was performed immediately following exercise, SpO_2_ had normalized in our subjects. While we would not expect changes in ventilation heterogeneity to recover immediately upon cessation of exercise, the study protocol would not have captured the presence of transient increases in ventilation heterogeneity that may have occurred during exercise. Furthermore, any delayed effect of exercise upon ventilation heterogeneity may have been mitigated by the administration of salbutamol as part of postexercise bronchodilator assessment. Finally, although there was considerable variation in flow rates across the three postexercise MBNW maneuvers, we have demonstrated that the variation in flow rates does not affect ventilation heterogeneity.

## Conclusions

In conclusion, we have demonstrated that ventilation heterogeneity is not increased following an incremental maximal exercise bout in healthy trained athletes. We have also demonstrated that the observed EIAH in healthy trained athletes is not a result of increased ventilation heterogeneity. Further study is required to investigate whether MBNW performed during exercise would reveal transient changes in ventilation heterogeneity.

## Conflict of Interest

None declared.
